# The distribution variation of pathogens and virulence factors in different geographical populations of giant pandas

**DOI:** 10.3389/fmicb.2023.1264786

**Published:** 2023-09-14

**Authors:** Mengyu Zhao, Yuxia Li, Wei Wei, Zejun Zhang, Hong Zhou

**Affiliations:** ^1^College of Life Sciences, China West Normal University, Nanchong, Sichuan, China; ^2^Liziping Giant Panda’s Ecology and Conservation Observation and Research Station of Sichuan Province, Nanchong, Sichuan, China; ^3^Shimian Agricultural and Rural Bureau, Shimian, Sichuan, China

**Keywords:** giant panda, different geographic populations, gut pathogens, virulence factors, metagenomes

## Abstract

Intestinal diseases caused by opportunistic pathogens seriously threaten the health and survival of giant pandas. However, our understanding of gut pathogens in different populations of giant pandas, especially in the wild populations, is still limited. Here, we conducted a study based on 52 giant panda metagenomes to investigate the composition and distribution of gut pathogens and virulence factors (VFs) in five geographic populations (captive: GPCD and GPYA; wild: GPQIN, GPQIO, and GPXXL). The results of the beta-diversity analyzes revealed a close relationship and high similarity in pathogen and VF compositions within the two captive groups. Among all groups, Proteobacteria, Firmicutes, and Bacteroidetes emerged as the top three abundant phyla. By using the linear discriminant analysis effect size method, we identified pathogenic bacteria unique to different populations, such as *Klebsiella* in GPCD, *Salmonella* in GPYA, *Hafnia* in GPQIO, *Pedobacter* in GPXXL, and *Lactococcus* in GPQIN. In addition, we identified 12 VFs that play a role in the intestinal diseases of giant pandas, including flagella, CsrA, enterobactin, type IV pili, alginate, AcrAB, capsule, T6SS, urease, type 1 fimbriae, polar flagella, allantoin utilization, and ClpP. These VFs influence pathogen motility, adhesion, iron uptake, acid resistance, and protein regulation, thereby contributing to pathogen infection and pathogenicity. Notably, we also found a difference in virulence of *Pseudomonas aeruginosa* between GPQIN and non-GPQIN wild populations, in which the relative abundance of VFs (0.42%) of *P. aeruginosa* was the lowest in GPQIN and the highest in non-GPQIN wild populations (GPXXL: 23.55% and GPQIO: 10.47%). In addition to enhancing our understanding of gut pathogens and VFs in different geographic populations of giant pandas, the results of this study provide a specific theoretical basis and data support for the development of effective conservation measures for giant pandas.

## 1. Introduction

In the conservation of natural world heritage, the giant panda is widely recognized as a crucial flagship species (Zhu D. et al., 2021). The gut microbiota plays a vital role in the growth, development, and immune processes of giant pandas (Zhu et al., 2011; Zhang et al., 2018; Zhu et al., 2018; Zhao et al., 2021) and is significantly influenced by various factors including diet, phylogeny, seasons, and habitat environment (Zhang et al., 2018; Tang et al., 2020; Hu et al., 2021; Mustafa et al., 2021; Huang et al., 2022). Despite the successful downgrading of the conservation status of giant pandas from endangered to vulnerable (Guo et al., 2019), their health and survival are threatened by many factors. Among these, intestinal diseases are one of the primary causes of mortality in giant pandas (Qiu and Mainka, 1993; Janssen et al., 2006; Zhao et al., 2021). *Escherichia coli*, *Klebsiella*, *Campylobacter jejuni*, *Arizona*, *Pseudomonas aeruginosa*, *Enterococcus hirae*, *β-hemolytic streptococci*, and *Clostridium welchii* have been identified as major opportunistic pathogenic bacteria responsible for inducing different intestinal diseases (e.g., diarrhea and gastrointestinal inflammation) in captive giant pandas (Sun et al., 2002; Zou et al., 2018).

Opportunistic pathogens are widely distributed in animal habitats and can enter the host gastrointestinal tract by multiple routes such as food, water sources, and the fecal–oral route (Archie et al., 2009; Natchu and Bhatnagar, 2013; Chiyo et al., 2014; Springer et al., 2016; Czepiel et al., 2019). Changes in gut microbiota can influence the resistance and pathogenicity of these pathogens (Bäumler and Sperandio, 2016). Captive and wild environments significantly impact the structure and function of gut microbiota in giant pandas (Guo et al., 2019; Yao et al., 2019; Tang et al., 2020; Hu et al., 2021; Cui et al., 2023). Furthermore, antimicrobial resistance genes (ARGs) also exhibit different distribution patterns among different geographic populations of giant pandas. Mainly, the Qinling population, which has experienced long-term geographical isolation, shows a higher abundance of *Clostridium* and vancomycin resistance genes (Hu et al., 2021). Antibiotic interference is one of the strongest factors affecting the composition of gut microbiota and the ecological niche of pathogens (Diard and Hardt, 2017), which can decrease the diversity of gut microbiota and result in an increase in the pathogen population (Khan et al., 2021). Moreover, the diversity of ARGs in the gut was positively correlated with the diversity of virulence factors (VFs) (Escudeiro et al., 2019; Dionisio et al., 2023). VFs are one of the crucial aspects of pathogen evolution (Diard and Hardt, 2017), assisting pathogens in evading or modulating host defense systems by enhancing their adhesion, motility, growth, and survival capabilities, among other characteristics, thereby promoting successful colonization and infection (Yang et al., 2015; Sicard et al., 2017; Jurado-Martín et al., 2021; Pakbin et al., 2021). Given the variations in the gut microbiota of giant pandas across different geographic populations, the composition of pathogens and their VFs may also differ according to geographic patterns.

Furthermore, the gut microbiota in humans holds potential as therapeutic targets for various diseases, such as cardiovascular disease, diabetes, and obesity (Su et al., 2022; Vijay and Valdes, 2022). In this study, we investigated the distribution of gut pathogens and VFs in five distinct geographic populations of giant pandas (GPCD, GPYA, GPQIN, GPQIO, and GPXXL) using published metagenomic data (Wu et al., 2017; Zhang et al., 2018; Zhu et al., 2018; Guo et al., 2019). By gaining further insights into the epidemiological characteristics and distribution patterns of pathogens, we can identify risk factors and strengthen disease monitoring and conservation management practices. Our results can provide foundational data support for the development of more effective strategies for the prevention and treatment of diseases in giant pandas.

## 2. Materials and methods

### 2.1. Data collection

We collected 52 metagenomes (raw data) of giant pandas from two captive populations (Yaan and Chengdu) and three wild populations (Qinling, Qionglai, and Xiaoxiangling Mountains): ten from the Yaan Research Base of the Wolong Research Center (GPYA) (Guo et al., 2019), seven from the Chengdu Breeding Center (GPCD) (Zhang et al., 2018), nine from the Qinling Mountains (GPQIN) (Wu et al., 2017), seven from the Qionglai Mountains (GPQIO) (Guo et al., 2019), and 19 from the Xiaoxiangling Mountains (GPXXL) (Zhu et al., 2018). Detailed information on the sample groupings and data is provided in Supplementary Table S1.

### 2.2. Raw data treatment and bioinformatics analysis

Trimmomatic was used to perform quality control of the raw reads (Bolger et al., 2014). This involved removing low-quality reads, eliminating adapter contamination, and discarding ambiguous bases. A sliding window approach with a size of 50 base pairs (bp) was implemented, moving from left to right along the sequence. If the average quality value within a window dropped below 20, the sequence was trimmed, starting from that window position and removing the subsequent right portion. After quality control, any remaining short sequences that fell below the 50 bp threshold were also eliminated. The sequence data was then aligned using the bioinformatics tool BWA-MEM to identify and eliminate contamination sequences originating from the putative host (Li, 2013). Clean reads were further assembled into contigs using Megahit (Li et al., 2016), and Salmon (Patro et al., 2017) was used to evaluate the quality of assembled sequences, discarding contigs with insufficient coverage. Gene prediction of contigs was performed using Prodigal (Hyatt et al., 2012), generating gene files for each metagenome. Subsequently, non-redundant (NR) gene sets were constructed using CD-HIT (Li and Godzik, 2006), ensuring an overlap of less than 90% and a shared sequence identity of less than 95% among the gene files. These gene profiles functioned as the basis for mapping the clean reads per metagenome to the pristine NR gene profile using Salmon (Patro et al., 2017) and determining the abundance (transcripts per million reads, TPM) for these NR gene profiles in each metagenome. Finally, a BLAST analysis of these genes was performed against the NCBI-NR database (Benson et al., 1999), and a customized program was used to generate a TPM abundance table for each species at different taxonomic levels.

Box plots were used to compare the relative abundance (TPM) of the top 10 opportunistic pathogenic bacteria among the five groups, and the statistical significance of mean differences among the groups was evaluated using analysis of variance at a predetermined significance level of *p* ≤ 0.05. Nonmetric multidimensional scaling (NMDS) was performed using Bray–Curtis distance (Beals, 1984) in the vegan package (Dixon, 2003) to analyze the abundance (TPM) of pathogens and VFs and identify potentially distinct clusters among the five geographic populations of giant pandas. Furthermore, the linear discriminant analysis (LDA) effect size (LEfSe) method (Segata et al., 2011) was used to assess the significant differences in gut pathogen abundance among the five groups. In addition, we used the Wilcoxon rank-sum test with a threshold set at an LDA score ≥ 3 and *p* ≤ 0.05 to evaluate the magnitude of significant differences.

### 2.3. Identification and annotation of gut pathogens and VFs

We screened the opportunistic pathogenic bacteria and their corresponding abundance (TPM) based on the pathogen catalog compiled from the multiple bacterial pathogen detection (MBPD) database (Yang et al., 2023). Subsequently, VFs were functionally annotated using the VF database (VFDB, http://www.mgc.ac.cn/VFs/) and the online analysis tool VFanalyzer (Liu et al., 2019). By using the annotation information on VFs from the VFDB database and the gene abundances, a customized program was used to accurately calculate the abundance (TPM) of each VF within the metagenome.

## 3. Results and discussion

### 3.1. Captive giant panda populations exhibit higher similarity in gut pathogen compositions and virulence factor profiles

**Figure 1:**
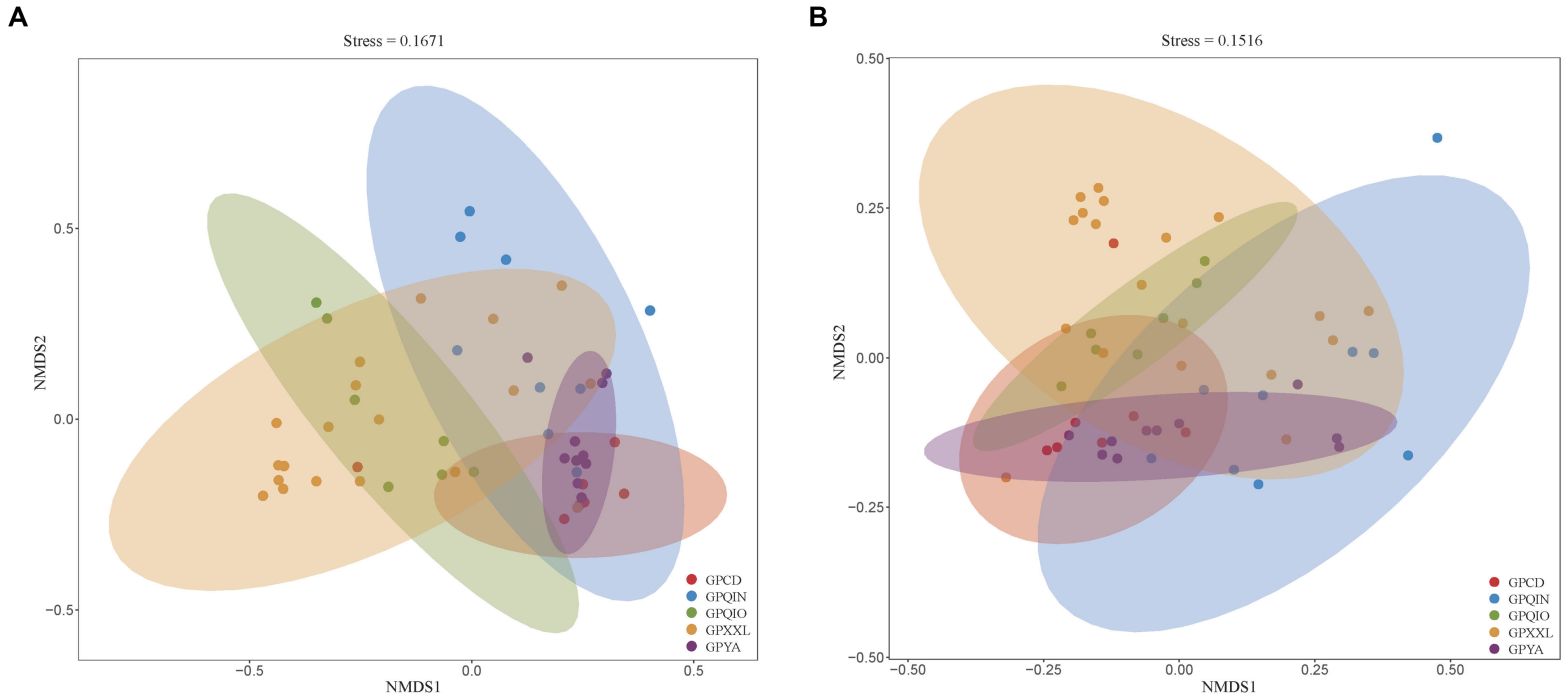
Nonmetric multidimensional scaling (NMDS) method based on Bray-Curtis distance showed the beta diversity in the composition of gut pathogens **(A)** and VFs **(B)** among five geographic populations of giant pandas. Nodes with different colors represent separate groupings. GPCD, the captive Chengdu giant panda population. GPQIN, the wild Qinling giant panda population. GPQIO, the wild Qionglai population. GPXXL, the wild Xiaoxiangling giant panda population. GPYA, the captive Yaan giant panda population.

We grouped samples according to the location of the geographic population of giant pandas (Supplementary Table S1) and compared their compositions of gut pathogens and associated VF profiles. NMDS analysis of all metagenomes of giant panda samples based on Bray–Curtis distance revealed distinct clusters, indicating potential dissimilarities. Notably, both clusters of gut pathogens (Figure 1A) and VFs (Figure 1B) exhibited close proximity in captive giant panda populations (GPCD and GPYA), forming tighter clusters compared with wild giant panda populations (GPQIN, GPQIO, and GPXXL). This was consistent with previous results observed in studies investigating the gut microbiota between captive and wild giant pandas (Guo et al., 2019; Yao et al., 2019; Hu et al., 2021; Cui et al., 2023).

### 3.2. The differences in gut pathogen compositions among the five geographic populations of giant pandas

**Figure 2:**
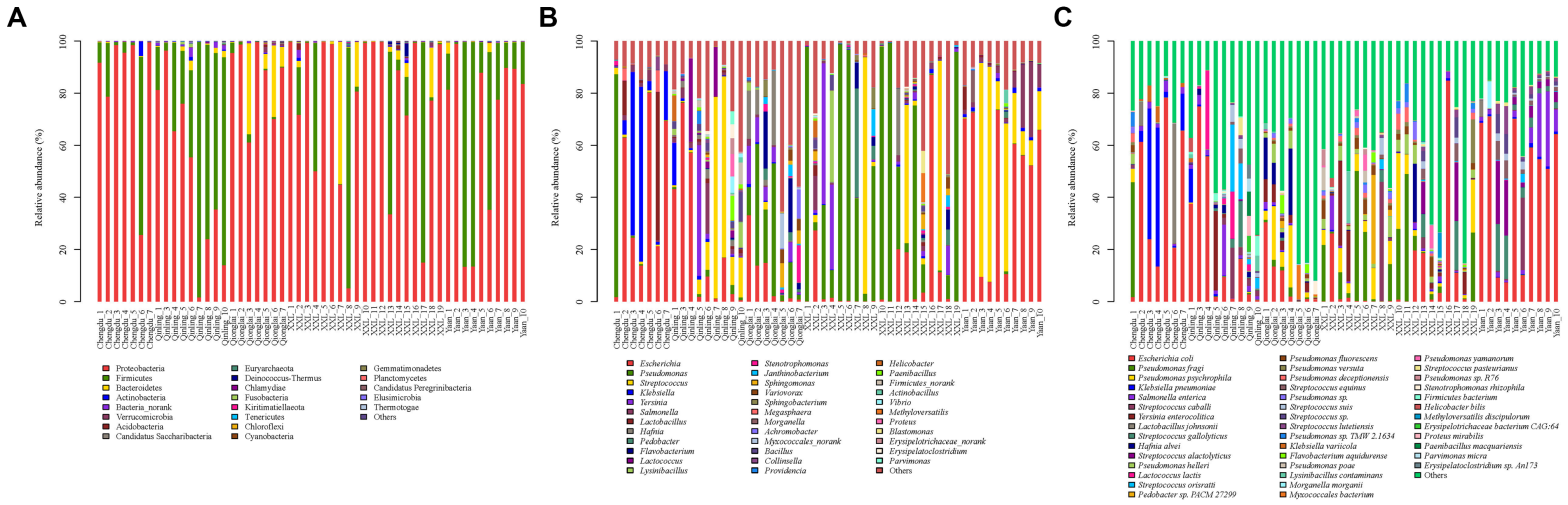
Composition of gut pathogens in each sample at the phylum **(A)**, genus **(B)** and species **(C)** levels. Different colors in the graphs depict distinct pathogen groups, with detailed information provided below each graph. Specific details regarding sample names are given in Supplementary Table S1.

The total sequences of 52 giant panda metagenomes were categorized into three main phyla (Figure 2A), including Proteobacteria (75.83%), Firmicutes (19.31%), and Bacteroidetes (4.1%), collectively constituting over 99% of the total abundance across all samples. Notably, Firmicutes (60.68%) exhibited the highest percentage in GPQIN, whereas Proteobacteria overwhelmingly dominated the other four groups (GPCD: 82.57%, GPYA: 72.80%, GPQIO: 86.92%, and GPXXL: 77.84%). At the genus level (Figure 2B), captive (GPCD and GPYA) and wild (GPQIN, GPQIO, and GPXXL) geographic populations of giant pandas exhibited distinct distribution patterns of gut pathogens. The most abundant taxon in captive populations (GPCD: 42.12% and GPYA: 53.07%) was *Escherichia*, whereas in the wild populations, *Pseudomonas* dominated in GPQIO (21.81%) and GPXXL (51.92%), and *Streptococcus* showed the highest proportion in GPQIN (39.66%). At the species level (Figure 2C), *Pseudomonas fragi* (15.69%), *E. coli* (10.19%), and *P. psychrophila* (7.45%) were the three most abundant species in GPXXL. However, *E. coli* overwhelmingly dominated the other four groups, with a relative abundance of 40.73% in GPCD, 51.63% in GPYA, 24.26% in GPQIN, and 8.92% in GPQIO. Furthermore, certain pathogen species exhibited significantly higher relative abundances in specific independent groups compared with the other groups, such as *Klebsiella pneumoniae* (16.00%) and *Lactobacillus johnsonii* (9.06%) in GPCD, *Salmonella enterica* (8.42%) in GPYA, *Lactococcus lactis* (14.23%) and *Streptococcus orisratti* (11.32%) in GPQIN, and *Hafnia alvei* (7.77%) in GPQIO. Notably, the captive population of giant pandas exhibited a higher prevalence of *E. coli* and *K. pneumoniae* compared with their wild counterparts. These two bacterial species isolated from the feces of captive giant pandas carry multiple antibiotic resistance genes and demonstrate resistance to various antimicrobial drugs, posing a serious threat to the health of captive individuals (Wang et al., 2022).

**Figure 3:**
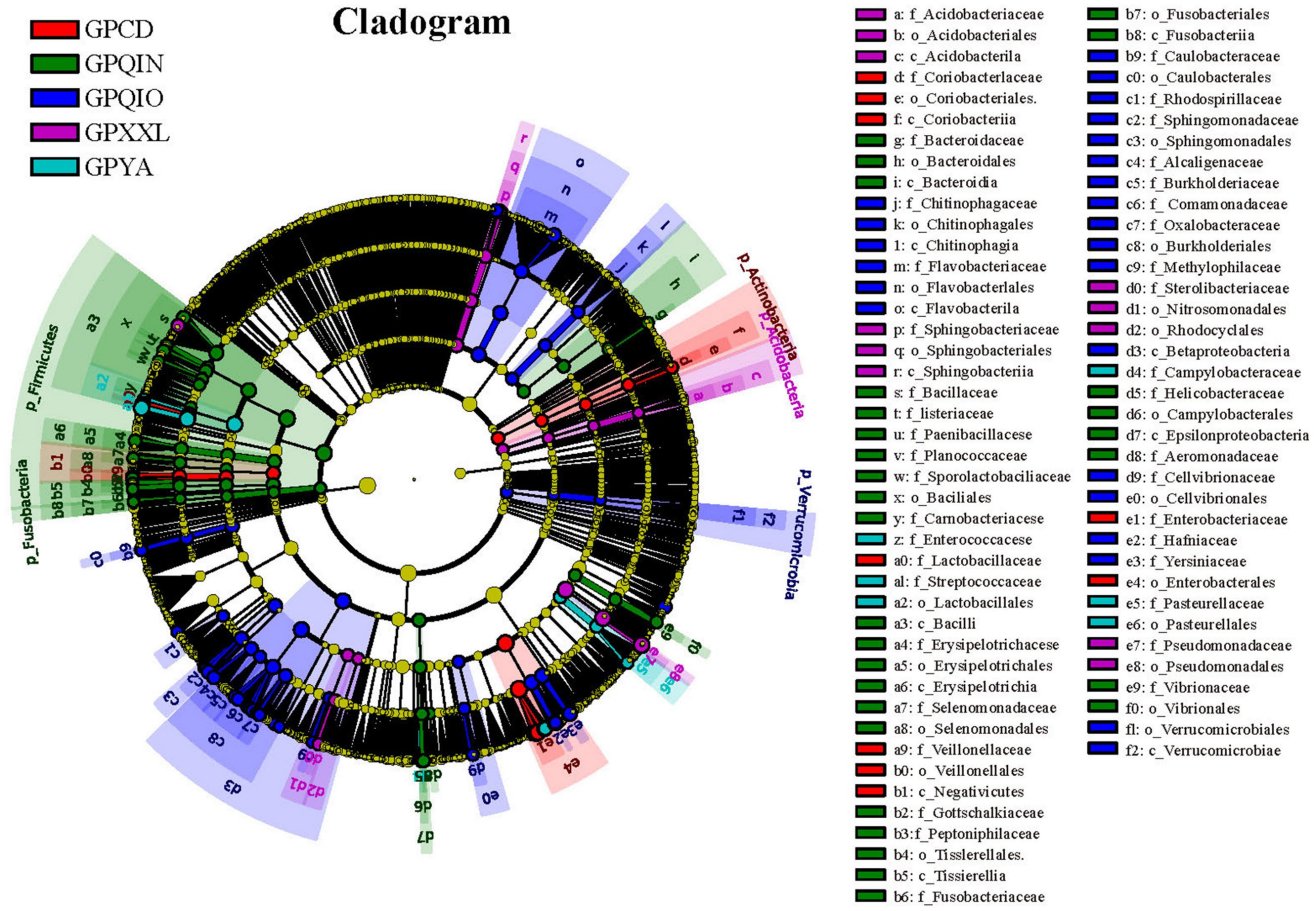
LEfse analysis of gut pathogens in giant pandas from five different geographical populations. Cladogram of gut pathogens: The circles, from inner to outer, represent the phylogenetic levels ranging from phylum to genus. Different colored areas denote distinct groupings, while different colored nodes indicate pathogen taxa that play significant roles within each group. Yellow nodes represent pathogen taxa that do not play an important role in any group. The diameter of the circles is proportional to the relative abundance. The legend on the right side of the figure displays the names of the pathogen corresponding to the letters indicated.

LEfSe identified 151 bacterial taxa associated with gut pathogens and revealed unique variation in the abundance of gut pathogens among the five groups (Figures 3 and Supplementary Figure S1). For instance, in the captive populations, GPCD exhibited a significantly higher abundance of *Klebsiella* (LDA score: 5.01, *p* < 0.001), *Lactobacillus* (LDA score: 4.80, *p* < 0.01), and *Megasphaera* (LDA score: 4.04, *p* < 0.05), while GPYA showed a greater presence of *Salmonella* (LDA score: 4.60, *p* < 0.01) and *Shigella* (LDA score: 4.08, *p* < 0.001). In the wild populations, GPQIO had more abundance of *Hafnia* (LDA score: 4.60, *p* < 0.01), *Yersinia* (LDA score: 4.50, *p* < 0.05), and *Flavobacterium* (LDA score: 4.50, *p* < 0.01). *Pedobacter* (LDA score: 4.16, *p* < 0.01) and *Lysinibacillus* (LDA score: 3.95, *p* < 0.01) demonstrated higher abundance in GPXXL. Lastly, *Lactococcus* (LDA score: 4.56, *p* < 0.001) and *Helicobacter* (LDA score: 3.71, *p* < 0.05) were significantly more abundant in GPQIN. Diet has profound effects on the host gut microbiota and its metabolites (Ley et al., 2008a,b; Bäumler and Sperandio, 2016; Guo et al., 2019; Wei et al., 2019; Zhu L. et al., 2021; Huang et al., 2022). A high-fiber diet can increase butyrate production, making animals more susceptible to Shiga toxin infection and related severe diseases (Zumbrun et al., 2013). Compared with wild giant pandas, which predominantly consume a natural bamboo diet rich in fiber, captive individuals typically also consume more starch-rich diets (mixed grains, corn, and other animal foods) (Guo et al., 2019). However, in this study, bacteria were most closely associated with Shiga toxin production (Zumbrun et al., 2013; Melton-Celsa, 2014), and both *E. coli* (Supplementary Figure S2B) and *Shigella* (Supplementary Figure S3), which are theoretically associated with low-fiber diets, exhibited high relative abundance in captive populations (GPCD and GPYA).

### 3.3. The profiles of VFs in the five geographic populations of giant pandas

**Figure 4:**
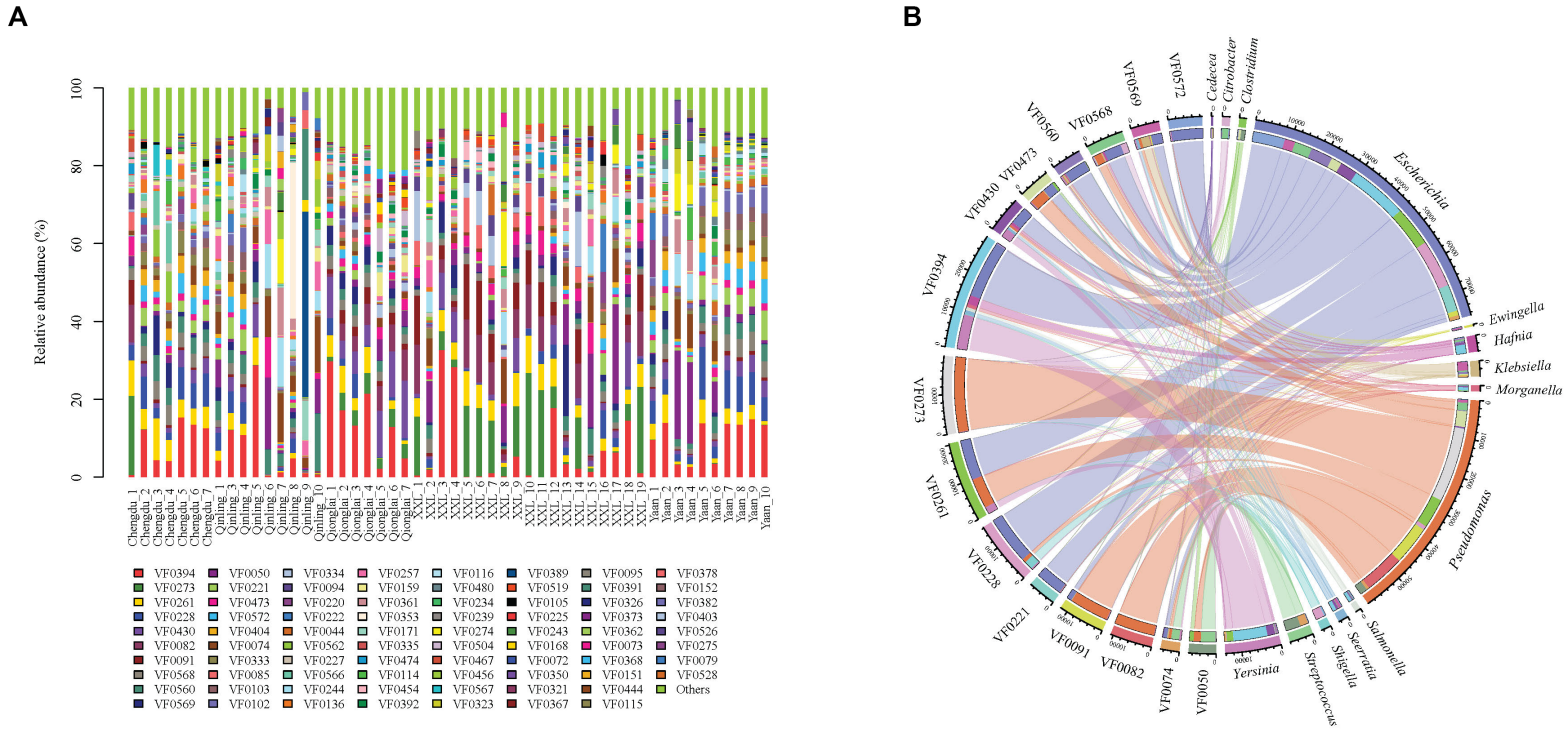
Composition of VFs in gut pathogens of giant pandas from five different geographical populations. **(A)** Different colors in the graphs depict distinct virulence factors, with detailed information provided below each graph. Specific details regarding sample names are given in Supplementary Table S1. **(B)** Composition of the top 15 VFs and the genera with the highest correlations to them.

Our metagenomic analysis identified 331 VFs and revealed their variance among five groups (Figure 4A). The top 15 most abundant VFs, including flagella (VF0394, VF0273, and VF430), CsrA (VF0261), enterobactin (VF0228), type IV pili (VF0082), alginate (VF0091), AcrAB (VF0568), capsule (VF0560), T6SS (VF0569), urease (VF0050), type 1 fimbriae (VF0221), polar flagella (VF0473), allantoin utilization (VF0572), and ClpP (VF0074), exhibited the highest correlation with 14 bacterial genera (*Escherichia*, *Pseudomonas*, *Yersinia*, *Streptococcus*, *Klebsiella*, *Hafnia*, *Serratia*, *Shigella*, *Salmonella*, *Morganella*, *Ewingella*, *Citrobacter*, *Clostridium*, and *Cedecea*) (Figure 4B) and nine species (*P. aeruginosa*, *Yersinia enterocolitica*, *Klebsiella pneumoniae*, *E. coli*, *Legionella pneumophila*, *Burkholderia pseudomallei*, *Helicobacter pylori*, *Aeromonas hydrophila*, and *Listeria monocytogenes*) (Supplementary Table S2).

In this study, we identified the potential of flagella, polar flagella, type 1 fimbriae, and type IV cilia to enhance pathogen movement, transmission, invasion, and colonization. A recent study conducted on gut pathogens in big-belly seahorse (*Hippocampus abdominalis*) also found that the flagella and type IV pili could promote pathogen motility, adherence, and invasion by enhancing the activities of key functional pathways associated with flagella assembly and bacterial chemotaxis within the gut microbiota (Zhang et al., 2023). Both ClpP (with the highest correlation to *L. monocytogenes*) and CsrA (with the highest correlation to *L. pneumophila*) were also identified as the prevailing VFs within the five groups. ClpP regulates the expression of CsrA at different growth stages of the life cycle of *L. pneumophila* by degrading accumulated CsrA protein and modulating the transcriptional expression of *csrA* mRNA, thereby ensuring the survival, proliferation, and pathogenicity of *L. pneumophila* (Ge et al., 2022). Other major VFs also enhance pathogens through different functional pathways. The capsule and alginate help pathogens in immune evasion and anti-phagocytosis. Enterobactin (iron uptake), urease (acid resistance), T6SS (antibacterial activity), AcrAB (antibiotic resistance), and allantoin utilization (provision of nitrogen source) also play a role in the survival, reproduction, and infection of pathogens. In this study, AcrAB (VF0568), strongly associated with *K. pneumoniae*, was abundantly expressed in the captive populations (Figure 4B). Overexpression of efflux pump genes results in multidrug resistance (Yerushalmi et al., 1995; Edgar and Bibi, 1997). In addition, this gene represents the predominant type of antibiotic resistance gene in *K. pneumoniae* strain K85 and serves as the primary determinant of bacterial resistance (Wang et al., 2022). The resistance genes present in the *K. pneumoniae* K85 genome were also associated with insertion sequences and integron–integrase genes (Wang et al., 2022), which may contribute to the further dissemination of antimicrobial resistance among captive giant pandas.

### 3.4. Differential expression of VFs in gut pathogens among the five geographic populations of giant pandas

VF0394 and VF0273 were the two most abundant components of VFs found in this study, with VF0394 exhibiting the strongest correlation with *Y. enterocolitica* and VF0273 with *P. aeruginosa*. Among the five geographic populations, VF0394 was the most abundant VF in GPCD (9.80%), GPYA (11.81%), GPQIN (8.88%), and GPQIO (17.27%), while in GPXXL, VF0273 (10.96%) was the predominant VF, followed by VF0394 (7.07%). The highest proportions of VF0394 in GPQIO and VF0273 in GPXXL were also consistent with the significantly abundant presence of *Yersinia* (LDA score: 4.50, *p* < 0.05) and *Pseudomonas* (LDA score: 5.36, *p* < 0.001) within their respective groups (Supplementary Figures S1, S2A). In addition, the distribution of VFs in the wild geographic populations displayed more distinct dissimilarity. VF0082 (with the highest correlation to *P. aeruginosa*) was more abundant in GPQIO (4.41%) and GPXXL (6.32%) compared with the other three groups (Supplementary Figure S4), and GPQIN, GPQIO, and GPXXL exhibited higher proportions of VF0074 (5.09%, with the highest correlation to *L. monocytogenes*), VF0430 (8.61%, with the highest correlation to *B. pseudomallei*), and VF0091 (6.27%, with the highest correlation to *P. aeruginosa*), respectively.

Among the nine pathogenic species with high virulence expression (Supplementary Table S3), *Y. enterocolitica* and *K. pneumoniae* exhibited consistently higher levels of virulence across all groups, whereas *E. coli* and *P. aeruginosa* showed a higher relative abundance of VFs in captive and wild giant panda populations, respectively. Notably, virulence levels of *P. aeruginosa* were different between the GPQIN and non-GPQIN wild populations. The lowest virulence level of *P. aeruginosa* was found in the GPQIN population (0.42%), whereas high virulence levels were observed in the non-GPQIN wild populations (GPXXL: 23.55% and GPQIO: 10.47%). Consequently, the long-term isolation of Qinling giant panda populations might not only contribute to variations in the composition of gut microbiota and ARGs (Zhu et al., 2011; Wu et al., 2017; Hu et al., 2021) but also impact the differential expression of VFs in gut pathogens.

## Data availability statement

The raw data supporting the conclusions of this article will be made available by the authors, without undue reservation.

## Ethics statement

The animal study was approved by the animal study was reviewed and approved by Research Ethics Review Committee of China west Normal University (CWNU2020D08). The study was conducted in accordance with the local legislation and institutional requirements.

## Author contributions

MZ: Investigation, Resources, Validation, Writing – original draft, Writing – review & editing. YL: Investigation, Validation, Writing – review & editing. WW: Formal analysis, Investigation, Software, Writing – review & editing. ZZ: Conceptualization, Software, Writing – review & editing. HZ: Conceptualization, Resources, Writing – original draft, Writing – review & editing.

## Funding

The authors declare financial support was received for the research, authorship, and/or publication of this article. This study was supported by the Science and Technology Department of Sichuan Province (2022JDR0033 and 2022JDJQ0060).

## Conflict of interest

The authors declare that the research was conducted in the absence of any commercial or financial relationships that could be construed as a potential conflict of interest.

## Publisher’s note

All claims expressed in this article are solely those of the authors and do not necessarily represent those of their affiliated organizations, or those of the publisher, the editors and the reviewers. Any product that may be evaluated in this article, or claim that may be made by its manufacturer, is not guaranteed or endorsed by the publisher.
