# Supplementary material for: The distribution variation of pathogens and virulence factors in different geographical populations of giant pandas
Source: Front Microbiol. 2023 Sep 14;14:1264786. doi: 10.3389/fmicb.2023.1264786 (PMC10543425; doi:10.3389/fmicb.2023.1264786)
Supplement: Supplementary file 1 [file Data_Sheet_1.docx]

# Supporting information

**Table S1 Information on the giant panda samples used in this study**

| **Sample ID** | **Analysis ID** | **Living style** | **Location** | **Citation** |
| --- | --- | --- | --- | --- |
| SRR5415505 | Yaan_1 | Captive | Yaan | (Guo et al., 2019) |
| SRR5130540 | Yaan_10 | Captive | Yaan | (Guo et al., 2019) |
| SRR5415504 | Yaan_2 | Captive | Yaan | (Guo et al., 2019) |
| SRR5150002 | Yaan_3 | Captive | Yaan | (Guo et al., 2019) |
| SRR5149981 | Yaan_4 | Captive | Yaan | (Guo et al., 2019) |
| SRR5149936 | Yaan_5 | Captive | Yaan | (Guo et al., 2019) |
| SRR5149935 | Yaan_6 | Captive | Yaan | (Guo et al., 2019) |
| SRR5149886 | Yaan_7 | Captive | Yaan | (Guo et al., 2019) |
| SRR5130583 | Yaan_8 | Captive | Yaan | (Guo et al., 2019) |
| SRR5130565 | Yaan_9 | Captive | Yaan | (Guo et al., 2019) |
| ERX2429102 | Chengdu_1 | Captive | Chengdu | (Zhang et al., 2018) |
| ERX2429101 | Chengdu_2 | Captive | Chengdu | (Zhang et al., 2018) |
| ERX2429100 | Chengdu_3 | Captive | Chengdu | (Zhang et al., 2018) |
| ERX2429099 | Chengdu_4 | Captive | Chengdu | (Zhang et al., 2018) |
| ERX2429098 | Chengdu_5 | Captive | Chengdu | (Zhang et al., 2018) |
| ERX2429087 | Chengdu_6 | Captive | Chengdu | (Zhang et al., 2018) |
| ERX2333382 | Chengdu_7 | Captive | Chengdu | (Zhang et al., 2018) |
| SAMC011136 | Qinling_1 | Wild | Qinling | (Wu et al., 2017) |
| SAMC011114 | Qinling_10 | Wild | Qinling | (Wu et al., 2017) |
| SAMC011133 | Qinling_3 | Wild | Qinling | (Wu et al., 2017) |
| SAMC011132 | Qinling_4 | Wild | Qinling | (Wu et al., 2017) |
| SAMC011131 | Qinling_5 | Wild | Qinling | (Wu et al., 2017) |
| SAMC011127 | Qinling_6 | Wild | Qinling | (Wu et al., 2017) |
| SAMC011121 | Qinling_7 | Wild | Qinling | (Wu et al., 2017) |
| SAMC011118 | Qinling_8 | Wild | Qinling | (Wu et al., 2017) |
| SAMC011115 | Qinling_9 | Wild | Qinling | (Wu et al., 2017) |
| SRR5149961 | Qionglai_1 | Wild | Qionglai | (Guo et al., 2019) |
| SRR5149913 | Qionglai_2 | Wild | Qionglai | (Guo et al., 2019) |
| SRR5149902 | Qionglai_3 | Wild | Qionglai | (Guo et al., 2019) |
| SRR5149862 | Qionglai_4 | Wild | Qionglai | (Guo et al., 2019) |
| SRR5130600 | Qionglai_5 | Wild | Qionglai | (Guo et al., 2019) |
| SRR5130560 | Qionglai_6 | Wild | Qionglai | (Guo et al., 2019) |
| SRR5130537 | Qionglai_7 | Wild | Qionglai | (Guo et al., 2019) |
| 20140128-zx-F03 | XXL_11 | Wild | Xiaoxiangling | (Zhu et al., 2018) |
| 20131124-ZX-F03 | XXL_12 | Wild | Xiaoxiangling | (Zhu et al., 2018) |
| 50 | XXL_13 | Wild | Xiaoxiangling | (Zhu et al., 2018) |
| 34 | XXL_14 | Wild | Xiaoxiangling | (Zhu et al., 2018) |
| 31 | XXL_15 | Wild | Xiaoxiangling | (Zhu et al., 2018) |
| 9 | XXL_16 | Wild | Xiaoxiangling | (Zhu et al., 2018) |
| TT2 | XXL_1 | Wild | Xiaoxiangling | (Zhu et al., 2018) |
| 19 | XXL_10 | Wild | Xiaoxiangling | (Zhu et al., 2018) |
| TT1 | XXL_2 | Wild | Xiaoxiangling | (Zhu et al., 2018) |
| LZP | XXL_3 | Wild | Xiaoxiangling | (Zhu et al., 2018) |
| LX3 | XXL_4 | Wild | Xiaoxiangling | (Zhu et al., 2018) |
| CDXM40 | XXL_5 | Wild | Xiaoxiangling | (Zhu et al., 2018) |
| CDXM39 | XXL_6 | Wild | Xiaoxiangling | (Zhu et al., 2018) |
| CDXM27 | XXL_7 | Wild | Xiaoxiangling | (Zhu et al., 2018) |
| CDXM18 | XXL_8 | Wild | Xiaoxiangling | (Zhu et al., 2018) |
| 23 | XXL_9 | Wild | Xiaoxiangling | (Zhu et al., 2018) |
| 1 | XXL_17 | Wild | Xiaoxiangling |  |
| 6 | XXL_18 | Wild | Xiaoxiangling |  |
| 8 | XXL_19 | Wild | Xiaoxiangling |  |


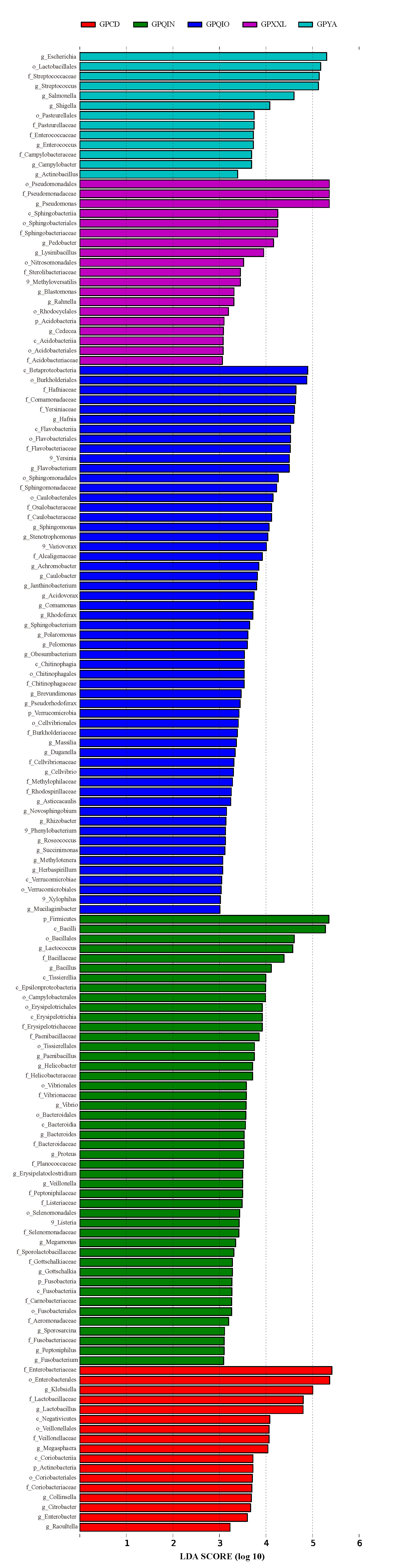


**Figure S1 The histogram of the linear discriminant analysis effect size (LEfSe) analysis was constructed to demonstrate the gut pathogens taxa with significant effects in each group.** The corresponding groupings were represented by different colors, and the magnitude of the contribution of the differential pathogens was depicted by the length of the bar chart. The differential pathogens were selected based on an LDA score greater than or equal to 3 and a significance level (P-value) less than or equal to 0.05. GPCD, the captive Chengdu giant panda population. GPQIN, the wild Qinling giant panda population. GPQIO, the wild Qionglai population. GPXXL, the wild Xiaoxiangling giant panda population. GPYA, the captive Yaan giant panda population.


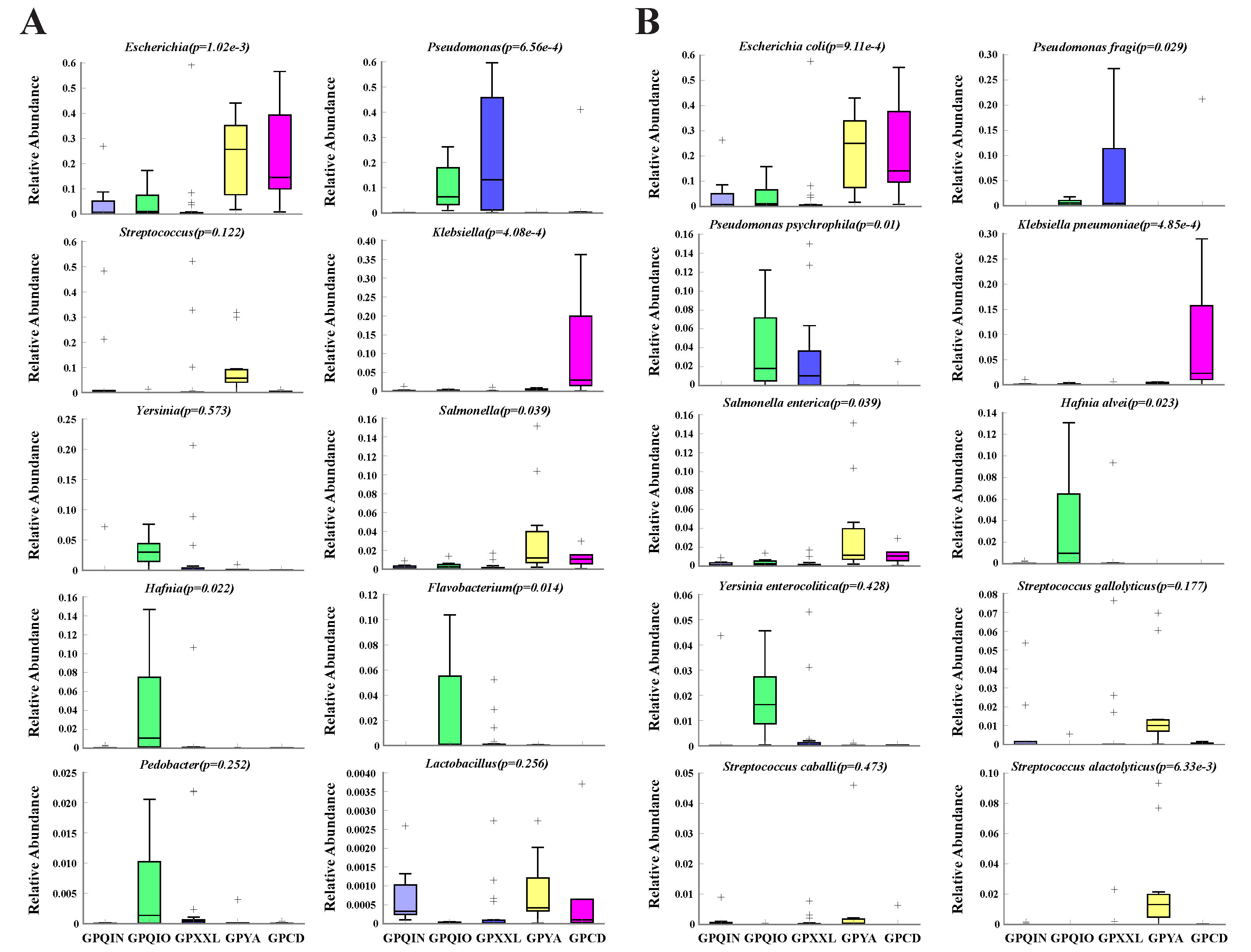


**Figure S2 Composition of the top 10 gut pathogens in giant pandas from five geographic populations at the genus (A) and species (B) level.** GPQIN, the wild Qinling giant panda population. GPQIO, the wild Qionglai population. GPXXL, the wild Xiaoxiangling giant panda population. GPYA, the captive Yaan giant panda population. GPCD, the captive Chengdu giant panda population.


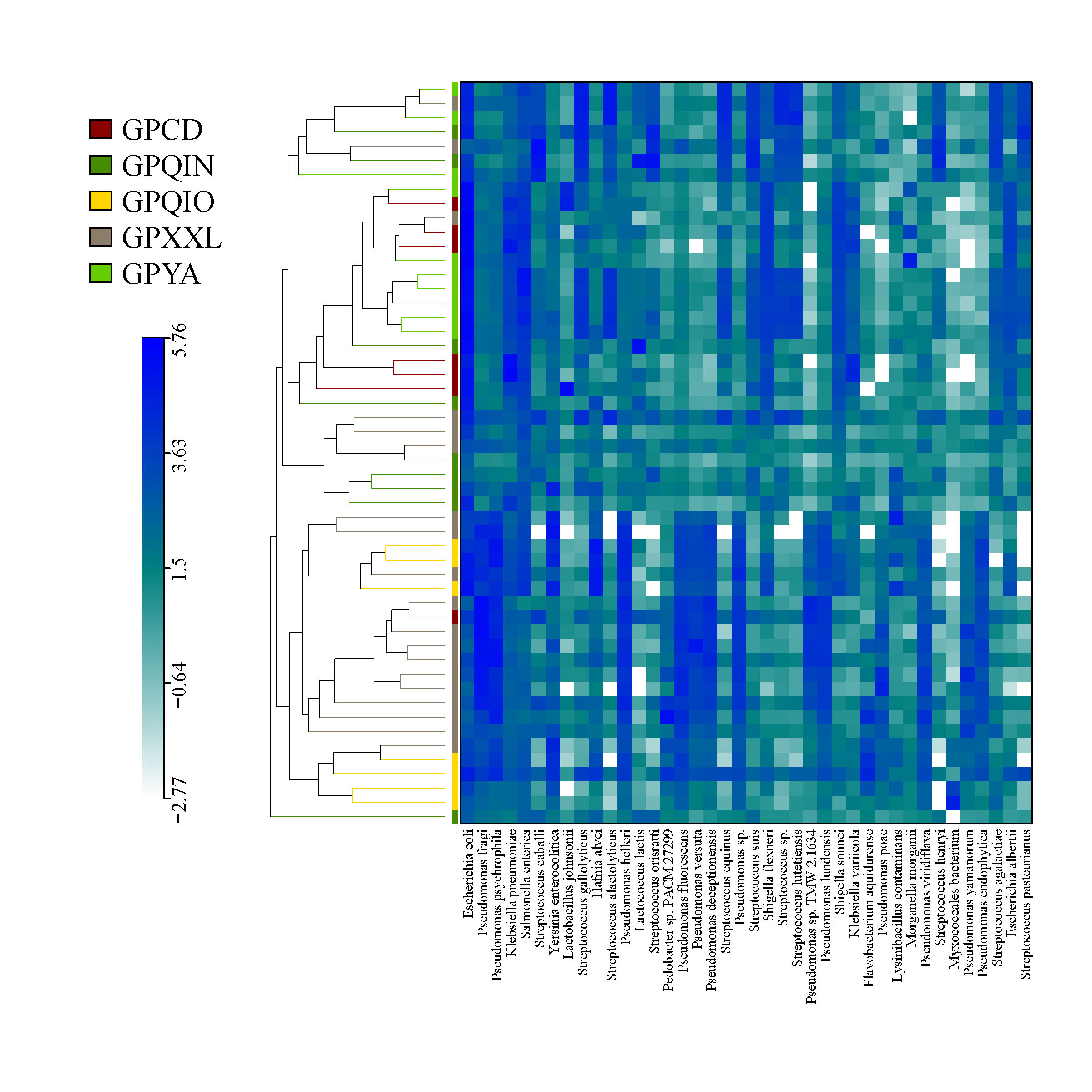


**Figure S3 Composition of the top 40 gut pathogens in giant pandas from five geographic populations at the genus level.** GPCD, the captive Chengdu giant panda population. GPQIN, the wild Qinling giant panda population. GPQIO, the wild Qionglai population. GPXXL, the wild Xiaoxiangling giant panda population. GPYA, the captive Yaan giant panda population.

## References:

Guo, W., Mishra, S., Wang, C., Zhang, H., Ning, R., Kong, F., Zeng, B., Zhao, J., and Li, Y. (2019). Comparative Study of Gut Microbiota in Wild and Captive Giant Pandas (Ailuropoda melanoleuca). Genes 10, 827. Krzywinski, M., Schein, J.E., Birol, I., Connors, J.M., Gascoyne, R.D., Horsman, D., Jones, S.J.M., and Marra, M.A. (2009). Circos: An information aesthetic for comparative genomics. Genome Research 19, 1639-1645.

Wu, Q., Wang, X., Ding, Y., Hu, Y., Nie, Y., Wei, W., Ma, S., Yan, L., Zhu, L., and Wei, F. (2017). Seasonal variation in nutrient utilization shapes gut microbiome structure and function in wild giant pandas. Proceedings of the Royal Society B: Biological Sciences 284, 20170955.

Zhang, W., Liu, W., Hou, R., Zhang, L., Schmitz-Esser, S., Sun, H., Xie, J., Zhang, Y., Wang, C., and Li, L. (2018). Age-associated microbiome shows the giant panda lives on hemicelluloses, not on cellulose. The ISME journal 12, 1319-1328.

Zhu, L., Yang, Z., Yao, R., Xu, L., Chen, H., Gu, X., Wu, T., and Yang, X. (2018). Potential mechanism of detoxification of cyanide compounds by gut microbiomes of bamboo-eating pandas. MSphere 3
